# Deconstructing the urinalysis: A novel approach to diagnostic and antimicrobial stewardship

**DOI:** 10.1017/ash.2021.167

**Published:** 2021-06-28

**Authors:** Sonali D. Advani, Christopher R. Polage, Mohamad G. Fakih

**Affiliations:** 1 Division of Infectious Diseases, Duke University School of Medicine, Durham, North Carolina; 2 Duke Infection Control Outreach Network, Durham, North Carolina; 3 Department of Pathology, Duke University School of Medicine, Durham, North Carolina; 4 Quality Department, Clinical & Network Services, Ascension Health, St. Louis, Missouri; 5 Wayne State University School of Medicine, Detroit, Michigan

## Abstract

The extensive use of the urinalysis for screening and monitoring in diverse clinical settings usually identifies abnormal urinalysis parameters in patients with no suspicion of urinary tract infection, which in turn triggers urine cultures, inappropriate antimicrobial use, and associated harms like *Clostridioides difficile* infection. We highlight how urinalysis is misused, and suggest deconstructing it to better align with evolving patterns of clinical use and the differential diagnosis being targeted. Reclassifying the urinalysis components into infectious and non-infectious panels and interpreting urinalysis results in the context of individual patient’s pretest probability of disease is a novel approach to promote proper urine testing and antimicrobial stewardship, and achieve better outcomes.

A perceived “abnormal” urinalysis result usually leads to the initiation of antimicrobials, often regardless of genitourinary symptoms.^1^ Early in training, most medical students and residents learn to view pyuria, bacteriuria, and infection interchangeably.^2,3^ Many clinicians order urine cultures and prescribe antibiotics inappropriately in asymptomatic patients with abnormal urinalysis parameters, often regardless of genitourinary symptoms^1^ contrary to national guidance.^4,5^ Recently, many US hospitals and medical centers have focused on reducing inappropriate urine cultures and leveraging the use of the urinalysis prior to obtaining a culture as diagnostic stewardship interventions.^6–9^ Such interventions have been influenced by biased economic incentives linked to catheter-associated urinary tract infection (CAUTI) prevention.^10^ Even though diagnostic stewardship interventions generally result in a reduction in the number of urine cultures ordered, their impact on appropriate antimicrobial use or clinician’s response to an abnormal urinalysis is not clear.

## Utility of urinalysis

The urinalysis is a popular screening test used across a wide range of inpatient and outpatient clinical settings, due to ease of accessibility, rapidity of results, and low cost. It is useful in the diagnosis and progression of wide range of medical conditions such as renal calculi, metabolic disorders, diabetes, acute and chronic kidney diseases, infections, stroke, andmalignancy.^11,12^ Urinalysis evolved over the last 200 years to include different chemical analyses and microscopic examination,^13^ making it a compilation of unaligned tests that only have their specimen source in common.^14^ Contrary to blood tests, where metabolic and hematologic panels are separately ordered, urinalysis does not have separate panels for workup of infection, metabolic disorders, or renal disease. As a result, physicians order a complete urinalysis for wide variety of reasons from general screening to cancer detection. Likewise, urinalysis is also overused to diagnose urinary tract infections in patients with nonspecific symptoms like confusion, fever, abdominal pain or sepsis without genitourinary symptoms. This has led to overuse of urinalysis in different settings, with 60%–80% of urinalyses being ordered in patients without symptoms referable to the genitourinary tract.^15–17^

## Urinalysis components

The different components of urinalysis, as it is done today, are described below:

1. *Gross examination of urine* includes description of color, odor, clarity, volume, and specific gravity. Urine color, clarity and volume may be altered due to many etiologies like dehydration, diet, medications, liver disease, infections, hematuria, and certain medical conditions.
2. *Chemical examination of urine* reflects parameters that may be encountered in a variety of acute and chronic illnesses. The urine dipstick is a rapid semiquantitative assessment of parameters such as pH, heme, albumin, specific gravity, glucose, leukocyte esterase, and nitrite.

Leukocyte esterase and nitrite have been traditionally used to evaluate for urinary tract infection (UTI). A positive test for leukocyte esterase may be seen in genitourinary inflammation, irritation from instrumentation or catheterization, glomerulonephritis, UTIs and sexually transmitted infections.^3^ Leukocyte esterase has a good negative predictive value but poor positive predictive value to diagnose infection.^18–20^ A positive test for nitrite can indicate presence of gram-negative bacteriuria, but it does not diagnose UTI in the absence of symptoms. Similarly, a negative test for nitrite does not rule out UTI, as some urinary pathogens like enterococcus do not produce nitrite. In addition, false-positive results for nitrite occurs on exposure to air or phenazopyridine, or from preanalytic contamination. As such, nitrite has poor sensitivity and specificity for diagnosing a UTI.^18^

- *Microscopic examination of the urine* enables confirmation of urine dipstick findings and also the identification of structures that are not evaluated by the urine dipstick (eg, epithelial cells, casts, crystals). It provides further information on inflammatory and non-inflammatory conditions. The presence of white blood cells (WBCs) in urine, also known as pyuria, is indicative of genitourinary inflammation. Pyuria occurs in 32% of young women, 90% of elderly patients in long-term care facilities, and 90% of hemodialysis patients with asymptomatic bacteriuria.^1^ Varying thresholds of pyuria [WBCs >5/high-powered field (hpf) vs WBCs >10/hpf] do not reliably predict bacteriuria or infection.^3,21^ Even though the absence of pyuria rules out infection, the positive predictive value of pyuria for identifying bacteriuria and UTI is low.^17,22^ Hematuria is also not a reliable predictor of infection. Red blood cells may be present in other medical conditions such as acute glomerulonephritis, stone disease, trauma, malignancy, or menstruation.^23,24^ On the other hand, large numbers of squamous epithelial cells (>5/hpf) may indicate a poorly collected sample. Renal epithelial cells may indicate renal tubular injury.^25,26^ Casts and crystals in urine may be benign or may represent underlying kidney disease (eg, nephrolithiasis, acute kidney injury) resulting from endogenous crystal production, exogenous drug exposure, inherited diseases, metabolic disorders, and/or drug exposure.^12^

The microscopic examination can also provide information on the presence of microorganisms in the urine but the clinical value has not been systematically studied and likely varies between populations.^27^ Most are performed by automated flow cytometry or image analysis with or without manual microscopic confirmation and cannot distinguish pathogens from nonpathogens or viable from nonviable organisms. Hence, detection of bacteria on the microscopic examination may be associated with positive urine cultures, but it cannot differentiate between asymptomatic bacteriuria, contamination, and UTI. Detection of yeast on the microscopic examination is usually secondary to colonization of urinary tract, indwelling catheter, or vaginal flora. Rarely, it may be due to a true yeast UTI, which is seen in neonates or patients with recent urologic instrumentation or surgery.^28^

## Misuse of urinalysis

Misuse of urinalysis can occur in all stages of testing: pre-analytic, analytic, and post-analytic phases. Misuse during the pre-analytic (ordering) phase occurs when urinalysis is ordered inappropriately for general screening or as a part of a noninfectious disease-specific workup. Urinalysis has been included in many screening and diagnostic protocols in emergency departments, medicine, pediatrics, nursing homes, outpatient clinics, and preoperative assessments.^29–32^ It is often bundled with other screening tests that are not related to an infectious diagnosis. Yin et al^15^ found that urinalysis was ordered in 62% of general medicine inpatients, but most of these patients (84%) were asymptomatic. In a national prevalence study of the urine testing, a urinalysis was ordered in almost half of the admissions.^33^ In another study of patients cared for in the emergency department, more than one-third of urinalyses were done without specific symptoms.^17^ There are also reports of monthly or quarterly standing urinalysis orders placed on nursing-home residents without any specific indications.^34^ A similar practice occurs in the ambulatory setting where urinalysis is ordered as an annual screening test (eg, order sets or potentially regular practices in an office for diabetics or psychiatric patients).^31,32^ Likewise, some surgeons order urinalysis or urine culture as a screening test in asymptomatic patients prior to joint replacement procedures based on expert opinion, low-quality evidence, and conflicting evidence.^16,30,35^

In the analytic phase, modifications to laboratory processing of urinalysis to reduce urine cultures may paradoxically lead to the misuse of urinalysis. For example, many US hospitals and laboratories use reflex urine cultures.^6,36,37^ In this approach, when a urinalysis is ordered, it automatically reflexes to urine culture when specific urinalysis parameters (eg, leukocyte esterase, nitrite, white blood cells, yeast or bacteria) are positive alone or in combination. These reflex algorithms became popular in the United States because of the emphasis on CAUTI prevention^6,9^ and the inclusion of CAUTI in the Centers for Medicaid and Medicare Services hospital-acquired–condition reduction program.^38^ Biased economic incentives have led to widespread adoption of reflex urine cultures in many settings.^39^ Although reflex urine cultures have resulted in a reduction in urine culture orders in patients without pyuria, this practice should be avoided in asymptomatic patients or those not suspected to have a UTI.^40^ Additionally, laboratories use different urinalysis parameters and cutoffs to proceed to culture, which leads to confusion and lack of standardized care.^36^ To complicate matters, different forms of urinalysis orderables exist within the same hospital as well as across multiple laboratories without guidance related to pretest probability or underlying diagnosis (eg, urinalysis macroscopic with reflex to microscopic urinalysis, complete urinalysis, urinalysis with reflex to culture, and/or urinalysis dipstick).^37^ Differences in laboratory processing and reporting of urinalyses make comparison of results across different hospitals, ambulatory clinics, and emergency departments impossible.^21^

In the post-analytic phase, screening for a medical condition using urinalysis may lead to unintended consequences based on incidental findings. The perception of abnormal results leads to further action from clinicians either following up with cultures or inappropriately treating with antimicrobials. For example, urinalysis performed in a diabetic patient for proteinuria may incidentally reveal pyuria or bacteriuria, which may trigger unnecessary urine cultures and or inappropriate antibiotic therapy. Patients with proteinuria may have concomitant asymptomatic bacteriuria, but they are not related.^41^ Clinicians, however, will often seize this abnormal result and prematurely mislabel the patient with a diagnosis of UTI.^40^

## Optimizing the urinalysis

Urinalysis and urine dipstick tests are easy and inexpensive screening tests, but their results can have important downstream consequences on urine cultures and antimicrobial prescribing. The level of pyuria on urinalysis correlates with increasing use of urine cultures and inappropriate antimicrobial prescribing.^1^ Gupta et al^1^ found that patients who were prescribed antimicrobial therapy for asymptomatic pyuria were not only unlikely to experience any reduction in risk of UTIs or surgical site infections, but also were more likely to develop adverse events like *Clostridioides difficile* infections. Due to its limited diagnostic utility, the Infectious Disease Society of America guidelines specifically recommend against using pyuria or bacteriuria as a criterion for the diagnosis of UTI or for administering antimicrobial therapy.^42^ Similarly, urinalysis results are not included in the National Healthcare Safety Network definitions of symptomatic UTI.^43^ Hence, diagnostic stewardship interventions should address precursor tests like urinalysis by uncoupling it from urine cultures, interpreting urinalysis results in the context of their pretest probability, and ideally, deconstructing urinalysis into components.

First, a concerted effort should be made to ensure that the urinalysis is only used when it provides significant value to manage a disease, regardless of whether it is an infectious or non-infectious condition. Routine urinalysis screening is a surprisingly common practice, used in ˜25% of emergency department visits, but does not directly impact decisions of care and delays the final disposition in most patients.^44,45^ Even though routine urinalysis testing (ie, screening) is presumed to help detect urinary tract malignancy, renal disease, and diabetes, these diseases are rare in young asymptomatic persons, making false-positive and incidental findings more likely. An annual urinalysis is not warranted for screening healthy asymptomatic individuals without major risk factors for bladder cancer (eg, persons with heavy exposure to cigarette smoke and other bladder carcinogens).^46,47^ Similarly, urinalysis has low utility in asymptomatic patients undergoing orthopedic, vascular, or cardiac surgeries.^29^ Hence, urinalysis should not be incorporated in general medical or surgical order sets unless it directly relates to the condition being managed.

Second, the value of urinalysis varies based on the patient characteristics and clinical scenario: catheterized versus noncatheterized patients, symptomatic versus asymptomatic patients, or older versus younger patients. For example, reflex urine cultures are useful when directed toward symptomatic noncatheterized patients, especially in the outpatient and emergency room settings. However, reflex urine cultures have poor utility in catheterized patients, neonates, and neutropenic patients.^40^ Clinicians should use the clinical context to develop a pretest probability for a likely diagnosis to which the urinalysis parameters should be applied. This process will allow the clinician to develop a differential diagnosis using the urinalysis parameters of significance while giving less weight to findings that are likely unrelated to the underlying kidney disease.

Third, we propose a novel diagnostic stewardship approach to consider various elements of urinalysis separately, based on the function they serve. Establishing panels based on common clinical indications for urine testing will allow clinicians to choose the panel that best aligns with their intended use and reduce unnecessary (off-target) testing. Currently, some hospitals limit the use of microscopy in patients that meet specific criteria on urinalysis, while other laboratories limit urine cultures to patients that meet specific urinalysis criteria.^27,48^ Laboratories can consider changing the ordering or reporting of urinalysis parameters to reflect underlying disease states based on the clinician’s evaluation: inflammation, metabolic disorders, renal disorders, etc (Table 1). Our suggested panels may serve as a starting point for streamlined evaluation of urine. These panels will require creating distinct orderables and reporting specific parameters based on pretest probability of disease. Making changes to urinalysis at the analytic stage may require manufacturing changes and US Food and Drug Adminstration reviews. An alternative intervention would be suppressing urinalysis components at the post-analytic phase. We encourage laboratories to evaluate their own data related to urinalysis so that these panels can be further refined based on institution specific ordering practices and needs. Using a directed approach to urinalysis would minimize identifying spurious results that may be detected as part of the bundled test of the urinalysis.

Lastly, for any of these interventions to be successful, they will need to be viewed as meaningful and necessary by adopters. More data are needed to evaluate the implementation of disease-specific urinalysis panels and any associated harms and benefits.^1,29,49^ The first step would be to pilot these directed urinalysis panels in certain settings and to evaluate the impact of these interventions on patient outcomes. Sustainability and long-term success will depend on the adaptive component of the intervention and organizational culture. For success in diagnostic stewardship interventions, both leadership and clinicians need to appreciate the ongoing value in these interventions.

In conclusion, the widespread indiscriminate use of urinalysis, especially as screening tests in emergency departments, clinics, hospitals and nursing homes, has led to serious downstream consequences. Abnormal urinalysis parameters in a patient without urinary symptoms is a powerful stimulus to order a urine culture and start antibiotic treatment, thwarting diagnostic and antibiotic stewardship interventions. A re-evaluation of the utility of the urinalysis and deconstructing the urinalysis to fit the diagnostic needs for patient care are critical first steps in mitigating the unnecessary urine cultures, inappropriate antibiotic use, and potential harms.

## Figures and Tables

**Table 1.** Urinalysis Components That Can Be Ordered In Lieu of Complete Urinalysis (UA)

Component	UA Inflammation	UA Metabolic	UA Renal	Complete UA (For Reference Only)
Gross evaluation		Color, odor, clarity	Color, odor, clarity	Color, odor, clarity
Chemical analysis	Leukocyte esterase	pH, albumin, specific gravity, glucose, bilirubin, ketones	pH, heme, albumin, specific gravity, glucose, bilirubin	pH, heme, leukocyte esterase, nitrite, albumin, specific gravity, glucose, bilirubin, ketones
Microscopic	White blood cells		White and red blood cells, casts, crystals	White and red blood cells, bacteria, yeast, epithelial cells, casts, crystals
